# HSP60 – a double edge sword in autoimmunity

**DOI:** 10.18632/oncotarget.5869

**Published:** 2015-09-28

**Authors:** Dorit Landstein, Rina Ulmansky, Yaakov Naparstek

**Affiliations:** ProtAb Ltd., Department of Medicine, Hadassah University Hospital, Jerusalem, Israel

**Keywords:** HSP60, autoimmunity, cytokines, therapeutics, Immunology and Microbiology Section, Immune response, Immunity

Human autoimmune inflammatory diseases are usually characterized by a chronic course. Antigen-induced experimental animal models for these diseases, on the other hand, typically present an acute or sub-acute course and after spontaneous recovery the animal may become resistant to reinduction of the disease. One explanation for this course of events may be that the antigen that induces the disease has a dual effect; on one hand it may serve as the target for the pathogenic immune attack whereas on the other it may lead to the development of protective immune mechanisms. Deficiency in these protective immune pathways may lead to the development of chronic autoimmune diseases. In the editorial below we will address this phenomenon by describing an example of one such antigen, HSP60 that has been shown to have a dual role in immune-mediated disorders, being involved in the induction and propagation of autoimmune diseases as well as in suppressing them [1].

HSP60 is a highly conserved protein, which was initially characterized as a mitochondrial chaperone. Upon exposure to stress or immune activation, HSP60 is also found in the cytosol, cell surface, extracellular space and biological fluids. HSP60 activates innate and adaptive immune responses, and can function as an endogenous danger signal to the immune system. It binds to cell surface receptors, including CD14 and members of the TLR family [reviewed by 1].

Overexpression of HSP60 has been described in tissues of various inflammatory diseases suggesting a possible pathogenic role of this antigen in their pathogenesis. Increased expression of human HSP60 is observed in intestinal ulcer of Behcet's Disease (BD) [2], in inflamed bowel of Crohn's disease patients, in muscle tissue of juvenile dermatomyositis (JDM) patients, and in synovial fluid and synovial tissue of juvenile idiopathic arthritis (JIA) and Rheumatoid arthritis (RA) patients [3]. HSP60 is also overexpressed in inflamed lesions of atopic dermatitis where it is co-localized with CD3 positive cells [4].

Peripheral mononuclear cells (PBMCs) taken from patients with various inflammatory diseases can be activated by HSP60. For instance, HSP60 induces IFNγ secretion from atopic dermatitis patients' PBMCs [4] and PBMCs of patients with BD, stimulated with various epitopes of HSP60, resulted in excess amounts of Th1 cytokine production [2].

Several supporting evidence for the pathogenicity of HSP60 have also been reported in experimental animal models of inflammatory diseases. HSP60 is upregulated in animal model of glomerulonephritis (NTN) as shown by increased HSP60 secretion in the urine and into the extracellular space of the diseased kidney. In addition, administration of HSP60 aggravates the disease in the NTN model and in experimental autoimmune uveitis in mice [5, 6].

Contrary to its role as a target for pathogenic autoimmune inflammatory processes, HSP60 has been shown to activate immunoregulatory pathways that may lead to suppression of these diseases. The finding that immunization with whole MT HSP60 prior to induction of adjuvant arthritis (AA) leads to protection against the disease attributed many experiments aimed at revealing the mechanism of this phenomenon, using mammalian and MT whole protein or synthetic peptides of the proteins. HSP60 based treatment ameliorated or suppressed several animal models of inflammatory diseases such as AA, collagen induced arthritis, insulin-dependent diabetes mellitus and atherosclerosis. Preventive as well as therapeutic HSP60 peptide treatment included various regimens, modes of administration and adjuvants and resulted in suppression of inflammatory cytokines, elevation of Th2 cytokines, and induction of regulatory T cells (CD4+CD25+Foxp3+) [3].

Interestingly, the presence of antibodies against peptide 6, a specific peptide derived from MT-HSP60, generated resistance to the induction of AA. We have recently shown that Prozumab, a humanized mAb targeting HSP60, is effective in ameliorating animal models of autoimmune inflammatory diseases such as RA and IBD. *In vitro* studies performed on human PBMCs were also indicative of an anti-inflammatory effect of Prozumab: Treatment of naïve PBMCs resulted in suppression of anti CD3-induced INFγ and IL-6 secretion [7].

Immune reactivity against HSP60 has been found to have a disease suppressing effect in human autoimmune inflammatory diseases as well. The presence of self-HSP60-specific T cell responses in JIA patients correlates with a benign disease course. Self-HSP-specific T cell responses have also been reported to be immunoregulatory in various other autoimmune diseases, such as RA and JDM, by the production of anti-inflammatory cytokines such as interleukin (IL)-10, IL-4 and TGF-β [3]. A phase 2 clinical trial testing the effect of Diapep277 (a human HSP60 derived peptide) in type 1 diabetes, has shown promising results. A phase 3 trial is currently ongoing [1, 3].

In summary HSP60 has been identified as a target for both pathogenic as well as protective immune mechanisms in many experimental and human inflammatory diseases. The upregulation of this protein at the sites of inflammation on one hand and its ability to skew the immune system towards suppression of disease on the other hand may explain the spontaneous remission of many experimental models of these inflammatory diseases. Understanding the putative mechanisms of disease suppression in animal models may lead to novel immunotherapeutic approaches to human autoimmune inflammatory diseases.
